# An App-Based Just-in-Time Adaptive Self-management Intervention for Care Partners (CareQOL): Protocol for a Pilot Trial

**DOI:** 10.2196/32842

**Published:** 2021-12-09

**Authors:** Noelle E Carlozzi, Sung Won Choi, Zhenke Wu, Jennifer A Miner, Angela K Lyden, Christopher Graves, Jitao Wang, Srijan Sen

**Affiliations:** 1 Department of Physical Medicine and Rehabilitation University of Michigan Ann Arbor, MI United States; 2 Department of Pediatrics University of Michigan Ann Arbor, MI United States; 3 Department of Biostatistics School of Public Health University of Michigan Ann Arbor, MI United States; 4 Clinical Trials Support Office University of Michigan Ann Arbor, MI United States; 5 Department of Psychiatry University of Michigan Ann Arbor, MI United States

**Keywords:** caregivers, quality of life, spinal cord injuries, Huntington disease, hematopoietic stem cell transplantation, feasibility studies, self-management, mobile apps, outcome assessment, mobile phone

## Abstract

**Background:**

Care partners (ie, informal family caregivers) of individuals with health problems face considerable physical and emotional stress, often with a substantial negative impact on the health-related quality of life (HRQOL) of both care partners and care recipients. Given that these individuals are often overwhelmed by their caregiving responsibilities, low-burden self-management interventions are needed to support care partners to ensure better patient outcomes.

**Objective:**

The primary objective of this study is to describe an intensive data collection protocol that involves the delivery of a personalized just-in-time adaptive intervention that incorporates passive mobile sensor data feedback (sleep and activity data from a Fitbit [Fitbit LLC]) and real time self-reporting of HRQOL via a study-specific app called CareQOL (University of Michigan) to provide personalized feedback via app alerts.

**Methods:**

Participants from 3 diverse care partner groups will be enrolled (care partners of persons with spinal cord injury, care partners of persons with Huntington disease, and care partners of persons with hematopoietic cell transplantation). Participants will be randomized to either a control group, where they will wear the Fitbit and provide daily reports of HRQOL over a 3-month (ie, 90 days) period (without personalized feedback), or the just-in-time adaptive intervention group, where they will wear the Fitbit, provide daily reports of HRQOL, and receive personalized push notifications for 3 months. At the end of the study, participants will complete a feasibility and acceptability questionnaire, and metrics regarding adherence and attrition will be calculated.

**Results:**

This trial opened for recruitment in November 2020. Data collection was completed in June 2021, and the primary results are expected to be published in 2022.

**Conclusions:**

This trial will determine the feasibility and acceptability of an intensive app-based intervention in 3 distinct care partner groups: care partners for persons with a chronic condition that was caused by a traumatic event (ie, spinal cord injury); care partners for persons with a progressive, fatal neurodegenerative disease (ie, Huntington disease); and care partners for persons with episodic cancer conditions that require intense, prolonged inpatient and outpatient treatment (persons with hematopoietic cell transplantation).

**Trial Registration:**

ClinicalTrials.gov NCT04556591; https://clinicaltrials.gov/ct2/show/NCT04556591

**International Registered Report Identifier (IRRID):**

DERR1-10.2196/32842

## Introduction

### Background

Care partners (ie, informal family caregivers) of individuals with health problems are faced with considerable physical and emotional stress [1-25], often with a substantial negative impact on the health-related quality of life (HRQOL) of both the care partner [1-3,5,7,26-44] and care recipient [14,45-61]. Care partners may suddenly be thrust into this full-time role and are often unprepared. As responsibilities accumulate, they face emergent health risks, including anxiety, fatigue, isolation, sleep problems, and decreased physical activity. Indeed, there is growing recognition that these psychological, social, and physical risks inadvertently affect patient health and well-being (ie, outcomes) [47,53,54,56-59,62-64]. Thus, it is imperative to develop novel interventions to support care partners to ensure better patient outcomes.

Despite the growing awareness regarding the importance of caregiving with the aging US population and evolving health care system, very little action has been taken to understand and improve conditions for care partners [39,40,65]. Thus, family caregiving (ie, care partners) is an urgent public health issue. With a high risk for developing depression, insomnia, and stress-related disorders [21,36-38,66-69], care partners are an ideal population to target for early detection and intervention strategies to treat compromised well-being. Although psychoeducation, skills training, or therapeutic counseling interventions can be effective for care partners, these interventions require intensive time and face-to-face commitment (with trained personnel), which can be prohibitive for an individual who is already overwhelmed by existing caregiving responsibilities and unable to make time for self-care.

### Objectives

This pilot study is designed to examine the acceptability and feasibility of an intensive data collection protocol that involves the delivery of a personalized self-management intervention to promote care partner self-care. Care partners from 3 distinct groups will be examined: care partners for persons with a chronic condition that was caused by a traumatic event (ie, spinal cord injury [SCI]), care partners for persons with a progressive, fatal neurodegenerative disease (ie, Huntington disease [HD]), and care partners for persons with an episodic cancer condition that requires intense, prolonged inpatient and outpatient treatment (persons with allogeneic hematopoietic cell transplantation [HCT]). Care partners will be randomized to either the control group, where they will wear the Fitbit (Fitbit LLC) and provide daily reports of HRQOL over a 3-month (ie, 90 days) period (without personalized feedback), or the intervention group, where they will wear the Fitbit, provide daily reports of HRQOL, and receive personalized push notifications for 3 months. The intervention is a just-in-time adaptive intervention (JITAI), that is, an emerging intervention that uses real time data collection to inform and personalize the delivery of the intervention [70,71]. Studies in other populations, including cardiovascular disease, diabetes, mental illness, and smoking cessation, support JITAI’s efficacy in improving physical, mental, and social health outcomes [72-76]. We describe the design and protocol of this trial in the following sections.

## Methods

### Participants and Setting

#### Overview

Data collection will include a diverse sample of N=60-90 care partners (n=20-30 SCI, n=20-30 HD, and n=20-30 HCT care partners). A care partner is defined as a person who provides physical assistance, financial assistance, or emotional support and who is not a professional, paid caregiver. Participant recruitment and enrollment will take place at the University of Michigan. The study was designed to be fully remote, given the ongoing restrictions related to the COVID-19 pandemic.

#### Inclusion Criteria

Care partners must be (1) aged at least 18 years, (2) able to read and understand English, and (3) caring for an adult (aged ≥18 years) with medically documented HD, SCI, or HCT. Care partners must be providing some form of care to the person with HD, SCI, HCT. Specifically, care partners must indicate a response ≥1 on the following question:

On a scale of 0-10, where 0 is ‘no assistance’ and 10 is ‘assistance with all activities,’ how much assistance does the person you care for require from you to complete activities of daily living due to problems resulting from his/her HD/SCI/HCT? Activities could consist of personal hygiene, dressing and undressing, housework, taking medications, managing money, running errands, shopping for groceries or clothing, transportation, meal preparation and cleanup, remembering things, etc.

Care partners must also have access to necessary resources for participating in a technology-based intervention (smartphone or tablet and internet access) and be willing to use their personal equipment or internet for this study, including downloading the CareQOL app (University of Michigan) and the Fitbit app on their mobile device, and be willing to complete all study assessments for the duration of study participation. Care partners of persons with SCI must be caring for an individual who is ≥1 year post injury and has a medically documented injury. Care partners of persons with HCT must indicate that they are caring for an individual who is receiving, has received, or is scheduled to receive HCT.

#### Exclusion Criteria

Professional, paid care partners (eg, home health aide) will be excluded from this study.

### Recruitment and Screening

Care partners will be recruited through existing clinical databases [77], registries, and relevant patient clinics at the University of Michigan, as well as through a study posting on UMHealthResearch.org and outreach to relevant community groups and organizations. Care partners will be recruited directly or through the person for whom they provide care. Individuals interested in participating will be encouraged to ask questions about the study and their participation, and if they opt to enroll, they will provide informed consent before completing any study assessments.

### Study Design

This pilot trial will use a 2-arm, randomized controlled design. Each of the 60 to 90 care partner participants will be randomized to an active *JITAI* arm (n=10-15 care partners of patients with SCI, n=10-15 care partners of patients with HD, and n=10-15 care partners of patients with HCT) or to a control arm (n=10-15 care partners of patients with SCI, n=10-15 care partners of patients with HD, and n=10-15 care partners of patients with HCT). The random allocation of participants to the treatment or control arm establishes the basis for testing the statistical significance or difference between the groups. All participants (regardless of study arm) will complete a baseline assessment comprising several self-report surveys designed to evaluate sample characteristics (ie, demographic information, medical history data, and patient characteristics) and HRQOL (CareQOL measures, as well as other caregiving measures and measures about the functional capabilities of the person with HD, HCT, or SCI). This is followed by a 10-day run-in period to allow for the shipping time of the Fitbit and to provide the participants time to familiarize themselves with the study technology (Fitbit and CareQOL app) and procedures. This period will also allow the study team to troubleshoot any potential barriers or issues that arise before the official start of the home monitoring period. For those who are randomized to the JITAI group, it will also allow for data collection that can be used to inform the intervention messages once the home monitoring period begins. This will be followed by a 3-month (90 days) home monitoring period during which participants will wear a wrist-worn Fitbit (to continuously monitor physical activity and sleep), as well as complete daily real time ratings of HRQOL (ie, single-item assessments of caregiver strain, anxiety, and depression). Those randomized to the JITAI group will have a 50/50 chance of receiving notifications each day during the home monitoring period (intervention details provided below). At the end of months 1 and 2, all participants will also complete a longer battery of self-report surveys on HRQOL (also delivered via CareQOL app), and at the end of 3 months, participants will complete this longer survey battery plus a feasibility and acceptability questionnaire (again delivered via the app).

### Study Procedures

#### Overview

Unless they opt to use their own personal Fitbit, participants will receive a Fitbit for the collection of sleep and activity (steps) data and will download the Fitbit app and study app, CareQOL, on their personal mobile devices (iOS or Android). The CareQOL app will deliver ecological momentary assessments (EMAs) once per day, compile and display data (including those collected on the Fitbit), deliver study notifications, including messages for the participant to complete the daily EMA and other study surveys, and deliver the personalized study intervention prompts to the intervention group (JITAI group).

All study participants will complete 3 EMA questions daily on the CareQOL app. Each participant will be prompted by a push notification in a 5-hour window (based on participant preference) from the app to answer the questions. The EMA questions comprised 1 question on care partner strain (taken from the CareQOL Caregiver Strain item bank), 1 question on anxiety (taken from the Patient-Reported Outcomes Measurement Information System [PROMIS] Anxiety item bank), and 1 question on depression (taken from the PROMIS Depression item bank). These questions will be administered as a computer adaptive test such that each day’s item will be based on the previous day’s response. Questions are on a 5-point scale, with higher scores indicating more of the named construct.

**Figure 1:**
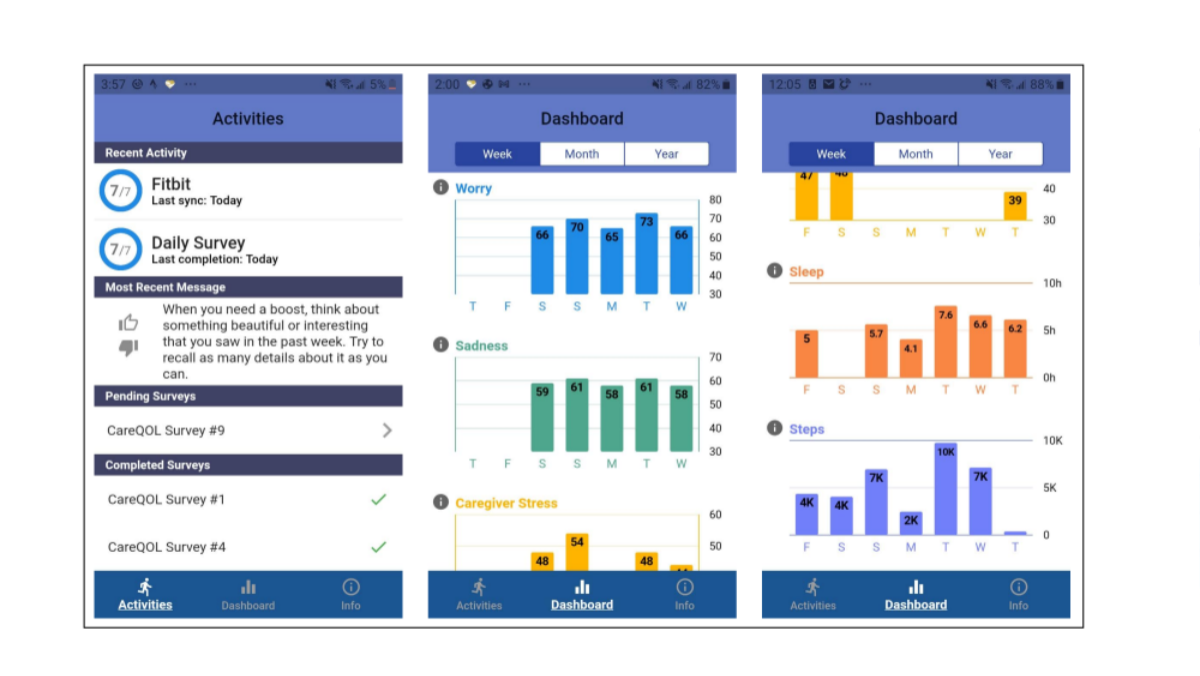
Screenshots of the CareQOL app.

In addition to the collection of EMA data, the app compiles and displays a graphical summary of historical data for care partner stress (strain), worry (anxiety), sadness (depression), steps (collected by the Fitbit), and hours of sleep (collected by the Fitbit) on a participant dashboard. Participants can view this information for the past week, month, or year (Figure 1). This is available to all participants as a pull—that is, it is available at all times but is accessed only if and when the user chooses to access it.

Care partners will be provided compensation for their participation in this study. Incentives will be identical for both groups (JITAI and control): US $20 compensation for the completion of the baseline assessment, US $10 for completion of each of the end-of-month assessments, US $1 per day for each day that they have EMA or Fitbit data during the home monitoring period, and the option to keep the Fitbit at the end of the study.

#### Randomization

Blocked randomization will be used to limit bias and achieve an equal distribution of participants to the control and treatment arms. A randomization list will be generated for each condition (SCI, HD, and HCT), and the study statistician will oversee randomization. The participants will be randomized once they are deemed eligible, have provided informed consent, and have completed the baseline assessments. The study coordinator or research assistant who consented the participants will use the appropriate condition’s randomization list to assign the participant to the correct study arm. Half of the participants will be randomized to receive the intervention (JITAI; described below); the remaining participants will be in the control group, who will not receive the JITAI but will complete the activities already described in this section. Participants who are randomized to receive the JITAI will have a 50/50 chance of receiving the intervention each day.

#### Intervention

The JITAI aims to promote behavioral change through motivational messages delivered through the CareQOL app as push notifications. These notifications provide a trigger for participants to initiate or continue behavior change or monitor behaviors (through engagement with the app); they are broadly based on the behavioral activation theory, which posits that negative life events (eg, difficult interactions between the care partner and care recipient and increased care partner stress because of caregiver role overload) trigger negative emotional responses (eg, depression and anxiety) that lead to unhealthy behavioral patterns (eg, poor sleep, decreased exercise, and social withdrawal), which starts the cycle all over again [78]. Specifically, the notifications are designed to foster care partner self-management by targeting behavioral change (ie, through increased physical activity and better sleep habits) and by promoting positive mental health responses (eg, self-efficacy, positive affect, and well-being). Behavioral activation (including behavioral activation delivered via SMS text messaging) is effective for treating both anxiety and depression as *pure* constructs and also for persons who are experiencing a mixture of the two [79-84].

The push notifications are low burden: participants can personalize the administration time (in a 5-hour window), and notifications can be viewed quickly on their phone’s lock screen. Participants can also choose not to engage with the notification at the time it is sent if it is inconvenient—they can return to it later if needed.

The JITAI push notifications are aimed at promoting healthy behaviors (physical activity and good sleep hygiene) and improving mood (anxiety, depression, and care partner strain). If a notification is to be sent, the content will be randomly drawn from this pool of messages. Some messages will use participants’ data directly in the messages (eg, you walked an average of 8120 steps this week), and most of the messages will be personalized based on data (eg, someone with low steps will get a different message than someone with medium steps than someone with high steps; high-medium-low). Messages comprise one or more of the following types: (1) data feedback, (2) facts, (3) tips, and (4) support. Table 1 provides specific examples of personalized push notifications that will be used in this study.

Randomization of the days the participants receive messages and the messages the participants receive from the pool will be done through the CareQOL app.

**Table 1:** Examples of personalized push notifications in the just-in-time adaptive intervention.

Feedback domain	Intervention options		
	Low level (below average performance or problems)	Medium level (average performance or problems)	High level (above average performance or problems)
Mental health (depression)	“Your average sadness rating over the last week was XX. Next time you’re feeling low, watch your favorite funny movie. Laughter is the best medicine!”	“Your average sadness rating over the last week was XX. When you’re feeling low, why not watch your favorite funny movie? Laughter is the best medicine!”	“Your average sadness rating over the last week was XX. If you’re ever feeling low, watch your favorite funny movie. Laughter is the best medicine!”
Mental health (anxiety)	“The next time you feel worried, close your eyes and think of a peaceful, relaxing place. Try to imagine as many different sights, sounds, and smells as you can. Continue until you feel more relaxed, then open your eyes slowly.”	“Are you feeling anxious? Close your eyes and think of a peaceful, relaxing place. Try to imagine as many different sights, sounds, and smells as you can. Continue until you feel more relaxed, then open your eyes slowly.”	“If you ever feel worried, close your eyes and think of a peaceful, relaxing place. Try to imagine as many different sights, sounds, and smells as you can. Continue until you feel more relaxed, then open your eyes slowly.”
Mental health (general)	“Is there a friend you haven’t talked to in a while? When you feel down, try giving them a call. Talking to friends can help boost your spirits!”	“Is there a friend you haven’t talked to in a while? Try giving them a call. Talking to friends can help boost your spirits!”	“Is there a friend you haven’t talked to in a while? If you feel down, try giving them a call. Talking to friends can boost your spirits!”
Mindfulness	“Take a few minutes every day to wind down. Even if you don’t feel stressed all the time, meditating can relieve built up tension.”	“Take a few minutes every day to wind down. Try meditating to relieve built up tension.”	“Take a few minutes every day to wind down. Even if you don’t feel stressed right now, meditating can relieve any built-up tension.”
Physical activity	“This past week, your average daily step count has been XX. Try to increase this if you can!”	“This past week, your average daily step count has been XX. Try to maintain this level, or even increase it more if you can.”	“This past week, your average daily step count has been XX. Great job! Try to maintain this level.”
Sleep	“You aren’t quite getting the recommended 7-8 hours of sleep per night. Try moving bedtime up by 5-10 minutes each night to get closer to this goal.”	“You’re having a hard time getting the recommended 7-8 hours of sleep per night. We all struggle to get to sleep sometimes. Try moving bedtime up by 5-10 minutes each night.”	“If you ever having a hard time getting the recommended 7-8 hours of sleep per night, try moving bedtime up by 5-10 minutes each night.”

### Outcomes

The primary objective of this study is to establish the feasibility and acceptability of our intensive data collection protocol. Table 2 provides a detailed summary of the study assessments, and Table 3 provides the schedule of activities.

The primary end point will examine survey responses on the feasibility and acceptability questionnaire designed to evaluate our intensive data collection protocol in the full sample. Secondary end points will include attrition and adherence estimates (again across the full sample). Exploratory analyses will be conducted to identify trends for an improvement in HRQOL scores (ie, group differences between the JITAI and control groups at the end of the 3-month home monitoring period, with the hypothesis that the JITAI group will report better outcomes than the control group). Exploratory analyses may also compare important subgroups (eg, care partner groups that differ by diagnosis [SCI, HD or HCT], relationship type [parent vs spousal care partners], sex [male vs female care partners], and according to the functional status of the person they are caring for). Exploratory analyses will use an intention-to-treat approach where the participant will contribute data to the arm they are randomized to, regardless of the amount of data contributed (ie, the duration of participation).

**Table 2:** Study assessments.

Outcome measure and description of outcome measure			Assessment schedule						
			Baseline		Daily^a^		1 and 2 months (30 and 60 days)		3 months (90 days)
**Demographic information**									
	Study-designed form used to capture demographic data, including age, gender, race, ethnicity, education, marital status, work status, COVID-19 history or status, care partner data, care recipient data, and caregiving demands	✓^b^							
**Care recipient medical record information**									
	Study-designed form with information about the person with SCI^c^, Huntington disease, or HCT^d^ for whom the care partner is providing care (eg, date of diagnosis, details of diagnosis, and disease stage or severity)	✓							
**Caregiver Appraisal Scale [85]**									
	47 items that assess positive and negative aspects of the caregiving role; 4 separate subdomain scores (perceived burden, caregiver relationship satisfaction, caregiving ideology, and caregiving mastery) can be calculated; higher scores indicate better functioning; reliability and validity supported [86]	✓							
**Self-report version of the United Huntington Disease Rating Scale Independence Scale [87]**									
	Care partner–reported rating provides an estimate of the current level of the independence for the person that they care for; this measure is rated from 1 to 100 in intervals of 5, with higher ratings indicating higher level of independence; reliability and validity supported [88]	✓							
**Supervision Rating Scale [89]**									
	Single rating that the care partner provides about the overall amount of supervision that the person they care for receives; ratings range from 1 to 13, with higher ratings indicating greater levels of required supervision; reliability and validity supported [89]	✓							
**TBI-CareQOL^e^ Caregiver Strain SF^f^** [90,91]									
	Assesses perceived feelings of feeling overwhelmed, stressed, and *beat-down* related to the care partner role; scored on a *t* metric (mean 50, SD 10), with higher scores indicating more strain; reliability and validity supported [90-92]	✓				✓		✓	
**TBI-CareQOL Caregiver-Specific Anxiety SF [90,93]**									
	Assesses care partner perceived feelings of worry and anxiety specific to the safety, health, and future well-being of the person with TBI^g^; scored on a *t* metric (mean 50, SD 10), with higher scores indicating more anxiety; reliability and validity supported [90,92,93]	✓				✓		✓	
**PROMIS^h^ Sleep-Related Impairment SF [94]**									
	Evaluates the effect of poor sleep on daytime functioning; scored on a *t* metric (mean 50, SD 10), with higher scores indicating more sleep-related impairment; reliability and validity supported [90,94-99]	✓				✓		✓	
**PROMIS Fatigue SF [95,100]**									
	Evaluates self-reported symptoms of fatigue, ranging from mild subjective feelings of tiredness to overwhelming exhaustion that may decrease one’s ability to perform activities of daily living; scored on a *t* metric (mean 50, SD 10), with higher scores indicating more fatigue; reliability and validity supported [90,95-98,101-107]	✓				✓		✓	
**PROMIS Anxiety SF [95,100]**									
	Assesses self-reported feelings of fear, anxiety, and hyperarousal; scored on a *t* metric (mean 50, SD 10), with higher scores indicating more anxiety; reliability, validity, and responsiveness supported [90,96-98,106,108]	✓				✓		✓	
**PROMIS Depression SF [95,100]**									
	Assesses self-reported feelings of sadness and worthlessness; scored on a *t* metric (mean 50, SD 10), with higher scores indicating more depression; reliability, validity, and responsiveness supported [90,96-98,106,108,109]	✓				✓		✓	
**PROMIS Anger SF [95,100]**									
	Assesses self-reported feelings of irritability and frustration; scored on a *t* metric (mean 50, SD 10), with higher scores indicating more anger; reliability, validity, and responsiveness supported [90,96,98,108]	✓				✓		✓	
**NIH^i^ Toolbox Self-Efficacy–General SF [110]**									
	Assesses self-reported confidence in the ability to successfully perform specific tasks or behaviors related to one’s overall functioning; scored on a *t* metric (mean 50, SD 10), with higher scores indicating more self-efficacy; reliability, validity, and responsiveness supported [110-112]	✓				✓		✓	
**Neuro-QoL^j^ Positive Affect and Well-Being SF [113]**									
	Assesses parts of an individual’s life that are related to overall life meaning and purpose, well-being, and satisfaction; scored on a *t* metric (mean 50, SD 10), with higher scores indicating greater satisfaction; reliability, validity, and responsiveness supported [113]	✓				✓		✓	
**NIH Toolbox Perceived Stress [110]**									
	Assesses an individual’s feelings about the nature of events and individual coping resources; scored on a *t* metric (mean 50, SD 10), with higher scores indicating more perceived stress; reliability, validity, and responsiveness supported [110]	✓				✓		✓	
**PROMIS Ability to Participate in Social Roles and Activities SF [95,100]**									
	Assesses involvement in one’s ability to participate in usual social roles and activities; scored on a *t* metric (mean 50, SD 10), with higher scores indicating more ability to participate; reliability, validity, and responsiveness supported [90,96,98,106,108]	✓				✓		✓	
**PROMIS Global Health v1.2**									
	10 items that assess overall physical, mental, and social health; scored on a *t* metric (mean 50, SD 10), with separate scores for physical and mental health (higher scores indicate better health); responsiveness and validity supported [114-118]	✓				✓		✓	
**COVID HRQOL^k^**									
	Single item that assesses how concerned the participant is about COVID-19; scores range from 0 to 10, with higher scores indicating greater COVID-19–specific concerns	✓				✓		✓	
**Single-item Caregiver Strain [90,91]**									
	Assesses perceived feelings of feeling overwhelmed, stressed, and *beat-down* related to the care partner role; scored on a *t* metric (mean 50, SD 10), with higher scores indicating more strain; reliability and validity supported [90-92]			✓					
**Single-item Anxiety [95,100]**									
	Assesses self-reported feelings of fear, anxiety, and hyperarousal; scored on a *t* metric (mean 50, SD 10), with higher scores indicating more anxiety; reliability, validity, and responsiveness supported [90,96-100,106,108]			✓					
**Single-item Depression [95,100]**									
	Assesses self-reported feelings of sadness and worthlessness; scored on a *t* metric (mean 50, SD 10), with higher scores indicating more depression; reliability, validity, and responsiveness supported [90,96-98,106,108,109]			✓					
**Fitbit-based estimate of physical activity**									
	Fitbit *off-the-shelf* summary physical activity data includes steps, sedentary behavior, and light, moderate, and intense activity			✓					
**Fitbit-based estimate of sleep**									
	Fitbit *off-the-shelf* summary data for total sleep time and time spent in each stage of sleep (awake, rapid eye movement, and light sleep)			✓					
**Medical history, medications, treatments, and COVID questionnaire**									
	Study-specific forms will be used to capture medical history and current treatments or management strategies (medication and nonmedication—eg, exercise and mindfulness) and COVID-19 history	✓						✓	
**Adverse event or status update**									
	Self-reported changes in mental or physical health							✓	
**Feasibility and acceptability questionnaire**									
	Assesses the experience of the participant with the study methodology and technology, including the CareQOL app, Fitbit, and the JITAI^l^. Items are scaled from 1 to 5 to indicate level of agreement, where *1* indicates *strong disagreement* and *5* indicates *strong agreement*							✓	
**Optional: semistructured interview (JITAI group only)**									
	Assesses participant experiences and perceptions of the intervention messages that they received from the CareQOL app							✓	

^a^Daily surveys will be administered through the run-in and 3-month home monitoring periods.

^b^Assessment performed.

^c^SCI: spinal cord injury.

^d^HCT: hematopoietic cell transplantation.

^e^TBI-CareQOL: Traumatic Brain Injury Caregiver Quality of Life measurement system.

^f^SF: short from.

^g^TBI: traumatic brain injury.

^h^PROMIS: Patient-Reported Outcomes Measurement Information System.

^i^NIH: National Institute of Health.

^j^Neuro-QoL: Quality of Life in Neurological Disorders.

^k^HRQOL: health-related quality of life.

^l^JITAI: just-in-time adaptive intervention.

**Table 3:** Schedule of assessments.

Assessments	Pre-enrollment	Enrollment, day 10	Approximately 3 months^a^			
			Run-in^b^, days –10 to –1	End of months 1 and 2		End of 3-month assessment, 90 (±7) days
				30 days	60 (±7) days	
SCI^c^, Huntington disease, HCT^d^ documentation	✓^e^					
Care partner eligibility	✓	✓				
Informed consent		✓				
Demographics and baseline survey		✓				
Caregiver Appraisal Scale		✓				✓
UHDRS^f^ Independence Scale		✓				
SRS^g^		✓				
Medical record confirmation CRF^h^		✓				
HRQOL^i^ measures		✓		✓	✓	✓
Fitbit and CareQOL app instructions		✓				
Randomization		✓				
JITAI^j,k,l^ and control home monitoring			✓	✓	✓	✓
Daily EMA^m,n^						
Feasibility and acceptability questionnaire						✓
Medications, therapies, medical history, and COVID-19		✓				✓
Adverse events reporting						✓
Optional: semistructured interview^o^						✓

^a^Individual participant duration may vary depending on when the participant completes health-related quality of life assessments.

^b^Approximately 10 days in duration, to include time for shipping and at least 3-4 days of data collection.

^c^SCI: spinal cord injury.

^d^HCT: hematopoietic cell transplantation.

^e^Assessment completed.

^f^UHDRS: United Huntington Disease Rating Scale.

^g^SRS: Supervision Rating Scale.

^h^CRF: Case Report Form.

^i^HRQOL: health-related quality of life.

^j^JITAI: just-in-time adaptive intervention.

^k^Active intervention, including personalized push notifications.

^l^Includes daily wearing the Fitbit for sleep and physical activity monitoring.

^m^EMA: ecological momentary assessment.

^n^For both active and control groups.

^o^Optional semistructured interview for the intervention group only; separate consent required.

### Data Collection, Storage, and Protection

This project uses multiple electronic data capture and management platforms, such as Research Electronic Data Capture (REDCap; Vanderbilt University), CareQOL, Qualtrics (Qualtrics), Fitbit, University of Michigan Health Information Technology and Services server, Google Cloud, and Amazon Web Services Cloud. All platforms are designed for human subject research and comply with federal and local data and information security practices. The study data entry and study management systems are secured and password-protected. At the end of the study, all study databases will be deidentified and archived securely at the University of Michigan.

### Sample Size Considerations

The main purpose of the current trial is to establish the feasibility and acceptability of an intensive data collection protocol to inform a larger, later-stage effectiveness study on the JITAI in care partners of persons with chronic medical conditions. Thus, this study is designed to provide a point estimate of the effect of the JITAI for the future large-scale trial. Given that there are no formal power analysis calculations for this type of analysis, we have based the proposed sample size on our previous experience conducting these types of trials. Specifically, we believe that approximately 50 participants will provide sufficient numbers and diagnostic diversity to evaluate the feasibility and acceptability of new mobile health apps. Thus, our proposed sample size of N=60-90 care partners (at least 30 per arm and 20 per care partner group) exceeds this estimate and should provide a reasonable range of scores on the HRQOL outcome measures to guide later phase trial work.

### Statistics

#### Sample Descriptive Data

Care partners in each study group (JITAI and control) will be compared descriptively according to the CONSORT (Consolidated Standards of Reporting Trials) guidelines [119]. We will use 2-tailed *t* tests and/or analysis of variance to examine group differences for continuous variables (eg, age and HRQOL outcomes). Chi-square or Fisher exact tests will be used to examine group differences for categorical variables (eg, care partner type [SCI, HD, and HCT], sex, ethnicity, race, education, marital status, and relationship to care recipient).

#### Primary End Point

We will generate frequency counts for each of the feasibility and acceptability questionnaire items (*note:* items are scaled from 1 to 5 to indicate the level of agreement, where *1* indicates *strong disagreement* and *5* indicates *strong agreement*). Descriptive statistics will also be calculated. We hypothesize that this intensive data collection protocol will be both feasible and acceptable for care partners (regardless of group assignment). Feasibility and acceptability will be measured by ≥80% of participants indicating that care partners either *agree* or *strongly agree* that the different study elements are feasible and acceptable.

#### Secondary End Points

Attrition will be reported as the fraction of participants who will complete the final assessment out of the total number of study participants who completed the baseline assessment. In addition, the completion rates for the EMA assessments, the Fitbit data (ie, steps and sleep), and the monthly surveys will be calculated. Descriptive statistics will be calculated for the missing data across the study. We hypothesize that this intensive data collection protocol will be both feasible and acceptable for care partners (regardless of group assignment). Specifically, we expect ≥80% of participants to complete the study, that ≤60% of participants will be missing data for the daily assessment questions, and ≥80% of participants will complete each of the end-of-month surveys.

#### Exploratory End Points

We will also conduct analyses to determine if care partners in the JITAI group have better HRQOL at 3 months relative to the control group after controlling for baseline scores. Specifically, we will conduct a series of analyses of covariance to determine if individuals in the JITAI group have significantly better (1) care partner strain (as measured by CareQOL Caregiver Strain), (2) depression (as measured by PROMIS Depression), or (3) anxiety (as measured by PROMIS Anxiety) at 3 months after controlling for baseline scores on each respective measure (eg, analyses looking at 3-month care partner strain scores will control for baseline estimates of care partner strain). In addition, for those participants who will complete the semistructured interviews, we will assess care partners’ perceptions and preferences related to the intervention prompts to allow for future adaptations that involve targeting, tailoring, and personalization of these prompts.

### Ethics and Dissemination

All study procedures will be conducted in accordance with the US Code of Federal Regulations (CFR) applicable to clinical studies (45 CFR part 46, 21 CFR part 50, 21 CFR part 56, 21 CFR part 312, or 21 CFR part 812) and research best practices. The study procedures have been approved by Institutional Review Boards of the University of Michigan Medical School (application approval HUM00184455 and registered with ClinicalTrials.gov [NCT04556591]). The study results will be reported according to the CONSORT 2010 guidelines and the 2013 CONSORT Patient-Reported Outcomes extension guidelines [120,121].

## Results

This study was funded in March 2020 and received institutional review board approval in August 2020. Participant recruitment for this trial began in November 2020 and was completed in June 2021. Dissemination of trial results is forthcoming. We expect to publish the results for the primary outcomes in 2022.

## Discussion

### Overview

The proposed study aims to investigate the feasibility and acceptability of an intensive data collection protocol that involves the administration of the JITAI intervention to care partners of persons with significant health conditions. This protocol provides a description of the design and methods of this randomized clinical trial.

Although interventions exist to help improve care partners’ HRQOL, they are typically time-intensive and expensive and have limited success in improving HRQOL in these individuals. Despite clear advantages in terms of convenience, reach, and scalability with using mobile technologies (including JITAIs) to support healthy behavior change, their clinical utility in care partners remains untested. Furthermore, although much research on care partners has focused on a one-size-fits-all approach to assessment and treatment, there is a growing body of evidence to suggest that although there are many commonalities in the care partner experience, there are aspects of care that are inherently unique to different care partner groups [122-124]. This study will provide preliminary data that will allow us to begin to explore the commonalities and differences among different populations, specifically care partners (1) caring for a person with a chronic condition that was caused by a traumatic event; (2) caring for a person with a progressive, fatal neurodegenerative disease; and (3) caring for a person with an episodic cancer condition that requires intense, prolonged inpatient and outpatient treatment. These diverse care partner groups not only allow for important between-population comparisons that can be used to inform future trial designs and maximize their impact but also maximize the generalizability to other care partner populations.

### Conclusions

In summary, at the conclusion of this project, we will have established the acceptability and feasibility of a low-cost, low-burden self-management intervention to improve HRQOL for care partners of persons across 3 diverse groups of care partners. Ultimately, these pilot data will also provide justification for a larger clinical trial designed to examine the effectiveness of a JITAI in a larger cohort of diverse care partners (ie, those caring for individuals who have survived a disabling traumatic event, are experiencing progressive neurological disease, or episodic cancer conditions). It is our hope that this work will ultimately lead to improved HRQOL of care partners and those they care for.

## Abbreviations

CFR: Code of Federal Regulations
CONSORT: Consolidated Standards of Reporting Trials
EMA: ecological momentary assessment
HCT: hematopoietic cell transplantation
HD: Huntington disease
HRQOL: health-related quality of life
JITAI: just-in-time adaptive intervention
PROMIS: Patient-Reported Outcomes Measurement Information System
REDCap: Research Electronic Data Capture
SCI: spinal cord injury
